# Cleansing and Cleanliness in Abdominal Surgeons’ Operations

**Published:** 1897-02

**Authors:** Lawson Tait

**Affiliations:** New York and Albany (*honoris causa*), L. R. C. P. Edin., etc., Birmingham, Eng. 7 The Crescent


BUFFALO MEDICAL JOURNAL.

Vol. XXXVI. FEBRUARY, 1897. No. 7.

### Original Communications.

### CLEANSING AND CLEANLINESS IN ABDOMINAL SURGEONS' OPERATIONS.

By LAWSON TAIT, M. D. New York and Albany (*honoris causa*),
L. R. C. P. Edin., etc.,
Birmingham, Eng.

A FEW DAYS AGO I read the detailed description of an operation for the removal of a bullet lodged in the brain, the operation being done by one of our best known European surgeons and a pronounced follower of the school of Lister. He first removed the dressing and exposed the scalp, which looked like a huge billiard ball, excepting for one ominous black spot where the bullet had bored its unkindly way. Then he took a scalpel which was dripping with antiseptic, a precaution which had not been taken with the foregoing bullet, and deftly incised the scalp, while almost all the time an assistant allowed a fine stream of warm water—sterilised by being boiled and allowed to cool to a safe temperature—to play from an irrigator upon the field of the operation ; then instrument after instrument was used through the track of the wound, all evidently the subject of fear for they all dripped with antiseptics, while the locus of the infected bullet was left to the prey of the germs which had been carried there and had been working about for forty-eight hours before the operation.

The operator had not read Mr. Leedham Grieve's interesting papers on how difficult, if not impossible, it is to sterilise the hands of the operator, for he consistently made no attempt in the direction, and yet he closed the wound with a parcel of cotton wool feebly impregnated with corrosive sublimate. He then addressed his surroundings on the marvelous results obtained in modern times by antiseptic surgery. In the museum of the Royal College of Surgeons is a large iron bar, technically known as a pumper, which went under the influence of a charge of gunpowder from below the chin of the user and straight upward through the head and out at the vertex, carrying into the wound a lot of germs and leaving them there. No sterilised water was used about the superficial wound and no corrosive sublimate was then employed (about the end of last century), yet the patient got well and remained so for years. The museums of this country and of others literally swarm with specimens which prove the receipt of serious cerebral injury with complete and permanent recovery, so that the belief now seemingly established in the minds of surgeons for the present moment, that a stream of sterilised water and a few grains of corrosive sublimate in the superficial dressings gives any greater security for recovery, has no foundation in fact, and is a mere temporary mental aberration.

Some few months ago I read a paper by the same surgeon that I have already quoted, on the subject of the influence of germs, and in the short space of a column and a half of an ordinary medical journal he used in thirteen instances such phrases as "it is now fully established," "it is beyond dispute," "it must be universally acknowledged," "Smith has proved," "Jones's remarkable observations have established," and "a complete result of Robinson's original researches we must believe," though in not a single instance would I, for one, admit anything in any one of the single assertions. It happens that those indicated as Smith, Jones and Robinson are three piquant contributors of papers on the application of the ever-advancing, ever-developing, ever-changing conclusions of the bacteriologists to the practical work of the surgeon; and such men, always anxious for second-hand novelties, forget in one week what they said the week before. In their writings it is an easy matter to picture in detail the extraordinary phases of the evolution of the practice and principle of Listerism, though it is only fair to say that I use Lister's name here with this qualification, that he, whilst the originator and still the chief advocate of the doctrines so various and so varying, is not responsible for more than about half the nonsense which has grown round the original antiseptic religion. There have been a large number of surgical "Pauls" who have freely disseminated perplexing epistles to the various surgical churches of the world, and thereby much and very acrimonious differences have arisen.

The antiseptic generation has now sped its cycle from 1866 to 1896, and we have come back to the figure of the clock at which we started. The time exactly embraces my own surgical life. In

Glasgow and in Edinburgh I saw patients die of the same terrible infliction, no matter what had happened to them. I saw removal of breasts die with a fatality which seemed to rival that of amputation at the middle of the thigh, and yet in my own practice during the cycle, out of many hundreds of cases of removal of breasts—how many I could not venture to guess—I think it pretty certain that the mortality has been a long way under 5 per cent., and probably has not been 1 per cent.; in fact, I can call to mind only two fatal cases. The crowded wards, the deficient ventilation, the one solution dish and the one sponge in each ward, the want of ordinary lavatory cleanliness, were the causes of the terrible results. The carbolic oil, the putty and the lac plaster may have had some countervailing influence under the circumstances of these horrible old pest-houses, but in practice outside such influences they were useless. These details, together with others which ended finally in the harmonious logic of the spray, marked the first epoch of the antiseptic cycle, a time during which it was devoutly believed that every germ was potent for evil and every resting spore was a surgical frost. Every germ, every spore, every scrap of harmless dust must, therefore, be submitted to a process of destruction by some potent chemical agent. This chemical agent was constantly changed. As soon as one was contrived, adopted, beloaded and trusted, it was found by some new observers to be wanting when weighed in the chemical balance. The chemical manufacturers were nearly wild, and many of them were ruined by the continual changes, and the antiseptic market rate for years was something more variable than that of African gold shares.

When the spray was introduced, I was led, by circumstances altogether outside my own conviction, to range myself once more as a follower of the chemical antiseptic school, though I did not for a moment forget or neglect my old methods. I performed a hundred consecutive ovariotomies with a full and complete adoption of all the antiseptic precautions of the Listerian school of the period, and I published a paper in the Transactions of the Royal Medical and Chirurgical Society contrasting the details of that series with those of a consecutive hundred in immediate opposition to them, and the contrast was not in favor of the antiseptic practice.

By this time I had become thoroughly persuaded as to the future progress of the antiseptic doctrines and practice, and I expressed my prophecy, which I called an experiment, though it was more of the nature of a satire. I went through all the ceremonious observances, with gradually diluted solutions, until I used nothing but boiled water, and then that was dispensed with. Finally, I used only ordinary tap water, and then I gave up the spray. This is precisely what has happened all round. The poisonous solutions were weakened bit by bit, the spray abandoned, with an expression of shame that it had ever been introduced ; rigorous hunting for germs was slackened and the antiseptic belief so modified that it was at last accepted that not every germ was hurtful, but only such as might yet be identified.

But, on the other side, we found the cubic space allowed to each patient rapidly increased, new hospitals were built and, above all, the segregation of surgical patients enormously advanced by the erection of cottage hospitals all over the country. For my own part, between 1878 and 1880, I secured an accommodation of about forty beds, for the most part the occupants of which had each a separate room ; in fact, practically they may be said to have all had separate rooms. The effect was at once apparent, for my mortality went down from about 30 per cent. to less than five, and I had long runs of fifty, sixty, eighty, and once as far as a hundred and forty-six consecutive operations without a death. Even hysterectomy, the most obstinate of all abdominal operations in yielding satisfactory results, has within the last ten years given me runs of thirty, forty and even forty-five consecutive successes. What are the explanations of all this ? The answer is, that though I cannot produce anything from which I can "absolutely prove" or "make it apparent beyond doubt," or anything of the cock-sure order, yet I can give basis conclusions which will be with difficulty upset, and those who neglect them will have to bear serious responsibility in the criticisms of the future.

The first conclusion at which I arrived concerning abdominal operations was, and it remains the strongest now, that the more the patients submitted to them are separated the better. For this purpose, and for the greater part of my practice, I adopted, as I have said, a room for each patient. Sometimes, with a press of work, I was tempted to "pack," as we used to call it, that is, put two patients in the same room, after the first six or seven days, but I had so frequently to regret this that I ultimately abandoned it.

I am quite sure that there is much truth in one conclusion I have often advanced, that time has much to do with what will happen in septic infection of an abdominal section. The fourth night is the critical night with all save hysterectomies, and with them that period is not to be so definitely fixed. If an ovariotomy is all right on the fifth morning, the chances of her going wrong are small indeed. But if you "pack" them they will have hematoceles, stitch abscesses, pulmonary complications, mumps and all sorts of secondary troubles in a proportion far greater than if you keep them absolutely one in each room. These complications don't affect the mortality much, but they prolong the convalescence in a fashion of the most annoying kind. This has been still more impressed on me during the last three years, in which I have far more widely adopted the plan of operating in the houses of the patients and leaving the subsequent treatment of them to their private medical attendant. This is now possible, seeing that I sedulously avoid anything in the shape of gratuitous work; whereas for more than twenty years I did not get pay of any kind for more than one-fourth of my clientele, and not more than costs out of pocket for one-tenth of them. I am now, therefore, working solely in a class where it is possible to have all that is requisite in the way of accommodation in the houses of the patients, and after the operation is over I am seldom required to see the patients again, recoveries are so little interrupted. All this experience points out to me the extreme importance of segregation, and the uniform results of the work at the Sparkhill hospital, not in the hands of one man, but in the hands of all to whom it has been intrusted, prove this as far as proof in surgery can go; for segregation is carried out there almost as completely as it can be carried out in the best houses, whilst the perfection of the sanitary arrangements provided by the committee of management are almost absurd in their completeness of detail.

All this and the necessity for it was enforced on my mind coincident with the complete banishment of any fear of germs, either wholesale or individual, though there still remained with me the wholesome dread of certain specific poisons, the nature of which I do not know, and I think I may safely say that their nature is equally unknown to everybody else. First of all of these, as deadly beyond all things known to me, is the poison begot in the peritoneum and uterus of the puerperal woman and in some cases which have died after abdominal section.

This brings me to speak of the third phase of the slowly developing Listerism or antiseptic doctrines of surgery where it had begun to call itself aseptic and to adopt some of the minor doctrines and practices which I have been preaching and practising since 1881. All that I have been saying up till now leads me to that condition which, after segregation, constitutes my second general condition essential for success in abdominal surgery and going a long way to explain our modern success. I mean cleanliness.

Cleanliness in surgery may be divided into general and specific. General cleanliness, such as close attention to cleaning wards and all in them, the cleanliness of all linen bedclothing, etc., and the personal cleanliness of the surgeon and the members of his staff, are matters I need not waste time over. The details of specific cleanliness are matters much more in need of discussion.

For a long time in the earlier part of my practice, I allowed all properly introduced and qualified practitioners to visit it, and they came in great numbers, chiefly from America, but I soon had to stop this kind of hospitality and to limit admission to such as came as serious-minded students prepared to see and understand what they saw. The reason for this was simple: the "globe-trotter" came and saw one or two operations and departed without understanding anything he had seen and, if he published, he perversely misrepresented the facts. To this misrepresentation is due two misstatements which even now appear at intervals in the medical journals of the continent and America. The first is that I really am a devoted disciple of the chemical germ destroyer school, but that I have some substance in use which I will not disclose. The second is that my secret is "water sterilised by boiling" and this ridiculous blunder recurred only a few months ago at one of the great congresses of America and, strange to say, from the mouth of one of my old pupils, Dr. Ricketts, of Cincinnati, who spent six months with me some years ago.

It is now fourteen years since I have used sterilised water for any purpose save to raise common tap water to the temperature required, so that the mixture would be probably five parts common tap or well water and one part water which had been boiled and possibly sterilised. The mixture would have a temperature of about 102°, for my hands will not stand much more with comfort. I have yet to learn that such a mixture deserves the name of "sterilised."

My attention to specific cleanliness is as close as can be given. It may be divided in between the instruments and hands of the operator and the abdomen of the patient.

I hail with great satisfaction all the wonderful inventions and devices of the modern operating theatre for securing cleanliness, for that cleanliness can be secured only by the work of women, and women in themselves have not the slightest idea of cleanliness save on the surface and unless they belong to the really well-educated classes. This is a fact which no one knows so well as he who has gone through the filthy drudgery of a gynecological out-patient department, where fifteen out of sixteen of the patients have lice or fleas upon them, and often both. This is the material from which the great bulk of the so-called trained nurses are obtained, and with their training, unless most especially well looked after, they alter their habits only in the sense of the smart cap and attractive uniform. They remain as dirty as ever, and it is therefore necessary to give them shelves and boxes of plate glass.

All instruments with sliding tubes, screw or Clendon joints ought to be abandoned, every joint should be capable of being unshipped, and after every operation every instrument used should be scrubbed with raw turpentine and a brush and then well washed with soap and water ; if this is done, simple immersion in cold tap water at the next operation is all that is wanted.

Sponges—ah ! they want a paper to themselves. They are, and ought to be, the terror of the operating surgeon, and I can't stay now to say what I have to say about them, save that new sponges are bad, old sponges dangerous, and that none of them should ever be boiled. The Americans will have it that I boil my sponges, but I never did such a thing but once, and that ruined the lot.

Now I come to the real subject-matter of my paper—the cleanliness and cleansing of the patients.

I see that a number of superstitious observances on this subject are still recommended, such as the application of an antiseptic pad to the abdominal wall for twenty-four hours before the operation, and the like.

I have never employed any such plans, being quite content with a soap and water washing of the skin to remove the dead fat and epithelium with which women are always coated and generally thickly. If there were any real poison in the skin no antiseptic pad would remove it in twenty-four hours. The real poisons known to me as absolute realities, those which I have spoken of as occurring in puerperal peritonitis, cannot be removed by any known germicide from the hands of the surgeon infected by them. Mr.

Leedham Grieves's experiments seem to show that it is impossible to cleanse the hands from the ordinary spores of decomposition, and yet we know that nowhere is epithelium reproduced and shed at so rapid a rate as it is on the hand. My knowledge of the terribly infective power of the puerperal poison, from my own experience and that of others, has been so emphatic and the lesson so disastrous, that I am persuaded that the poison, whatever it be, permeates at least the whole epithelial layer and cannot be got rid of save by efflux of time and skin, and that it is not safe for anyone so infected to operate till at least a fortnight or three weeks have elapsed. Have there not been lessons enough in the same direction by the spread of puerperal fever from the hands of the accoucheur ?

I am not alarmed by the conclusions to which Mr. Leedham Grieves's observations point, for I don't fear the ordinary germ poison at all. But still I take the precaution of keeping my nails short and clean and of washing my hands in raw turpentine the last thing before performing any operation, and then washing off the turpentine by ordinary soap and water. My reason for this may be seen in the simple experiment of washing the hands three or four times in the ordinary way and then in perfectly fresh water, repeating the process with the previous addition of turpentine. After this last water has stood for a few minutes there will be seen on its surface evidence of dirt of a very convincing kind. That dirt must be either in the clear turpentine or it must be a layer of dirt removed from the hands by the turpentine after having resisted the previous efforts with soap only. The latter conclusion is that accepted by me, and it accounts for my using turpentine to the patient's skin and my own, as well as to my assistant's in the rare cases where I need one.

All the other details of every operation performed by me are conducted, as I have said, by the use of plain cold water, taken immediately from the tap or well and raised when necessary to the desired temperature by the addition of the water from the kettle or boiler ; nothing whatever is added to that water for instruments or sponges.

The final cleansing, and I think by far the most important of the lot, is the cleansing of the abdominal cavity during and after operations.

7 The Crescent.

